# Identifying borderline traits in a Brazilian community sample using the Dimensional Clinical Personality Inventory 2 factors

**DOI:** 10.47626/2237-6089-2024-0871

**Published:** 2025-09-18

**Authors:** André Pereira Gonçalves, Lucas de Francisco Carvalho

**Affiliations:** 1 Instituto Multidisciplinar em Saúde Universidade Federal da Bahia Vitória da Conquista BA Brazil Instituto Multidisciplinar em Saúde, Universidade Federal da Bahia, Vitória da Conquista, BA, Brazil.; 2 Universidade São Francisco São Paulo SP Brazil Universidade São Francisco, São Paulo, SP, Brazil.

**Keywords:** Personality disorders, psychological assessment, dimensional model, screening test

## Abstract

**Objective:**

This study aimed to examine the discriminative capacity of the Dimensional Clinical Personality Inventory 2 (IDCP-2) factors for identifying individuals with elevated borderline personality disorder (BPD) traits within a Brazilian community sample while proposing an optimal cutoff score for distinguishing high BPD trait levels.

**Methods:**

The participant cohort consisted of 1,469 adults who completed assessments, including the Level of Personality Functioning Scale – Brief Form 2.0 (LPFS), the Personality Inventory for Diagnostic and Statistical Manual of Mental Disorders, 5th edition (DSM-5) (PID-5), the IDCP-2, and the Structured Clinical Interview for the Diagnostic and Statistical Manual of Mental Disorders, 4th edition (DSM-IV) – Personality Questionnaire (PQ-SCID-II). We categorized participants into three groups utilizing the traits outlined in the Alternative Model of Personality Disorders (AMPD) from DSM-5 Section III. Furthermore, latent profile analysis based on PID-5 facets revealed the existence of three empirically derived profiles.

**Results:**

Our findings demonstrate that IDCP-2 factors exhibited substantial discriminative power, marked by large effect sizes across most factors. To minimize false negatives, we suggest a conservative cutoff score of 22 as the most effective threshold for identifying individuals with high levels of BPD traits.

**Conclusion:**

The BPD score generated from IDCP-2 factors holds significant promise in clinical practice, offering valuable insights into a patient’s propensity to exhibit a BPD profile and providing a comprehensive clinical profile.

## Introduction

Borderline personality disorder (BPD) constitutes a multifaceted mental health disorder that exerts its impact across diverse domains of an individual’s life. These domains encompass intricate interpersonal relationships, occupational challenges, and a pronounced diminution of self-esteem.^1-3^ Recognizing the presence of BPD holds pivotal significance as an initial stride toward ameliorating quality of life for the afflicted individual, while concurrently enhancing prognostic trajectories. Moreover, a judicious focus on tailored interventions mitigates the personal toll and potentially alleviates the financial burdens accompanying a comprehensive therapeutic process.^4^ To this end, the Dimensional Clinical Personality Inventory 2 (IDCP-2) was constructed by researchers, signifying a noteworthy stride in this endeavor.^5^ The IDCP-2 is a self-report scale that assesses pathological traits, encompassing facets that align with BPD.^6,7^ The addition of a borderline cutoff score within an existing measure supplements the clinician’s discrimination in evaluating the clinical difficulties of individuals who have duly completed the IDCP-2. This addition thus expands the scale’s clinical utility, particularly accentuating its capacity to identify nuanced impairments such as propensities towards self-harm, impulsivity, feelings of emptiness, relational fragility, an intense apprehension of abandonment, emotional volatility, and compromised emotional regulation. Our study endeavors to examine the discriminatory capacity of the IDCP-2 factors for identifying individuals within a Brazilian community cohort who exhibit an elevation in prototypical BPD traits. Additionally, an empirical cutoff is proposed herein, posited to demarcate individuals demonstrating elevated BPD tendencies effectively.

### Theoretical background

Individuals diagnosed with BPD exhibit a pronounced and persistent pattern of functional instability, encompassing a constellation of facets such as difficulty in interpersonal relationships, self-harming behaviors, and self-concept distortions. This intricate presentation is further compounded by impulsive and risky actions that extend to oneself and others.^4,8-11^ Empirical insights gathered from prior investigations reveal that the prevalence of BPD in the general population ranges between 1.1 and 3%, underscoring its significance within the mental health landscape.^12,13^ However, this prevalence escalates in clinical cohorts, exhibiting a range of 10.2 to 35.6%, underscoring its heightened clinical salience.^14,15^ An intricate interplay emerges between BPD and suicidal tendencies, with a staggering 60% of diagnosed individuals presenting episodes of suicide attempts, and a critical 8% accomplishing the act of self-annihilation.^8,16,17^ Furthermore, the intricacies of BPD have relevance for issues of substance abuse and compulsive behaviors.^9,18-20^ The diagnosis of BPD is characterized by intricate comorbidities, linking its pathogenesis with other psychiatric illnesses.^21-23^

The diagnostic framework for BPD can be grounded in the Diagnostic and Statistical Manual of Mental Disorders, 5th edition, Text Revision (DSM-5-TR), specifically within the Alternative Model of Personality Disorders (AMPD), expounded in Section III.^8^ The AMPD integrates the traditional categorical approach with dimensional perspectives to evaluate BPD traits. A BPD diagnosis is underpinned by manifestation of self and interpersonal dysfunction, delineated as criterion A. Concurrently, criterion B entails high levels of specific pathological traits. The diagnostic threshold necessitates the individual to exhibit trait elevation in at least four of the seven delineated traits, including impulsivity, propensity for risk-taking, and a disposition towards hostility. Supplementary BPD traits in the DSM-5 encompass emotional lability, anxiety, separation insecurity, and an inclination towards depressive tendencies. Incorporation of the AMPD in the DSM-5 was a worthy advance engendered by assimilation of insights from taxometric investigations. These studies showed a latent dimensional construct underlying BPD and other personality disorders, thus rationalizing the significance of embracing a dimensional framework.^24,25^

In addition to the DSM-5 AMPD framework, empirical research centered on the dimensional perspective highlights distinctive traits characteristic of BPD, encompassing Impulsiveness, Risk-Taking, Hostility, Emotional Lability, Anxiety, Separation Insecurity, Depressivity, Irresponsibility, and Deceitfulness.^26-28^Recent advances in structuring the mental disorder taxonomy are evident in the Hierarchical Taxonomy of Psychopathology (HiTOP).^29^ This framework proposes that BPD is comprised of pathological traits organized within two broad domains labeled Internalizing and Antagonistic Externalizing spectra. Internalizing denotes a disposition toward experiencing negative affect and mood disorder symptoms.^29^ Antagonistic Externalizing pertains to maladaptive interpersonal relationships driven by heightened antipathy, conflict, and a capacity for intentional harm without accompanying guilt.^30^ The HiTOP model enumerates BPD’s characteristics as emotional instability, anxiety, separation insecurity, hostility, fragility, avoidance of abandonment, and vulnerability.

The methodology for assessing characteristic BPD traits involves a two-step approach, commencing with screening and trait mapping, followed by an elaborate clinical interview performed by a proficient clinician.^31,32^ Self-report scales are commonly employed for initial screening and trait mapping, among which, the IDCP-2^7^ is a fine example.

The IDCP-2 is a self-report scale designed to assess pathological traits, drawing from DSM-5 Sections II and III for personality disorders. It is widely used in Brazilian scientific literature^33^ and adheres to international guidelines^34^ for psychological assessment and psychometric criteria. Comprising 210 items categorized into 47 factors and 12 higher-order dimensions, the IDCP-2 reflects concordance with contemporary concepts of mental disorder classification (e.g., HiTOP). Previous investigations have demonstrated the validity of IDCP-2 factors, including those encapsulating the fundamental traits of BPD.^35-37^

Research endeavors have been undertaken to assess the discriminative efficacy of IDCP-2 factors in identifying individuals with BPD.^7,38^ Specifically, Carvalho and Pianowski^7^appraised the discriminatory potential of IDCP-2 factors for BPD, revealing Hopelessness, Vulnerability, Anxious Worry, Impulsiveness, and Risk-Taking as optimal discriminators for BPD traits. In a related study, Carvalho and Pianowski^38^ sought to distinguish BPD from bipolar disorder, highlighting elevated BPD scores within the Vulnerability, Anxious Worry, and Hopelessness factors relative to the bipolar cohort. However, these findings warrant interpretation with caution due to methodological constraints. Notably, the absence of comparative scales assessing pathological traits to gauge the discriminative prowess of the IDCP-2, a desirable approach,^39,40^merits consideration. The studies’ samples, comprising fewer than 350 participants, also impose limitations on the scope of inference derivable from their outcomes. Furthermore, certain participants responded to a version preceding the IDCP-2, the IDCP, characterized by different item sets used for score computation. The researchers employed an equating procedure to merge the IDCP versions, potentially introducing substantial measurement error.^41,42^

We endeavor to enhance prior investigations employing the IDCP.^27,38^ To the best of our knowledge, this study is the first to use the IDCP-2 without relying on statistical methods to fill in missing cases (e.g., equating procedures), encompassing a substantial sample from the general population, and incorporating comparative data from external measures. Our objectives involve examining the capacity of IDCP-2 factors to discriminate individuals with heightened BPD typical traits within a Brazilian community sample. Moreover, we also seek to derive a composite score based on IDCP-2 factors and offer a cutoff to pinpoint individuals exhibiting marked BPD tendencies.

We compared IDCP-2 outcomes with findings derived from the Structured Clinical Interview for the Diagnostic and Statistical Manual of Mental Disorders, 4th edition (DSM-IV) – Personality Questionnaire (PQ-SCID-II), focusing on items relevant to BPD criteria. We anticipate all BPD-associated factors assessed by the IDCP-2 will contribute substantially to identifying elevated BPD traits, aligning with established literature.^8,26,27,29^ Furthermore, we posit that the foremost discriminative factors will include Vulnerability, Impulsiveness, Risk-Taking, Anxiety, and Depressivity, as derived from prior research.^7,26-29,38,43^

## Methods

### Sample and procedure

The initial sample consisted of 2,187 Brazilian adults recruited by convenience specifically for this study. We collected data over the internet using Google Forms and shared links on Facebook, WhatsApp, and Instagram. The study procedures complied with the provisions of the Declaration of Helsinki regarding research involving human participants (World Medical Association [WMA]) and were approved by the Research Ethics Committee of Universidade São Francisco (CAEE: 09117719.0.0000.5514).^44^ All participants digitally consented to data usage. The online survey conformed to the recommended standards for conducting and reporting web-based surveys, the Checklist for Reporting Results of Internet E-surveys (CHERRIES).^45^ The inclusion criterion was age ≥ 18 and education to at least elementary school. To ensure the quality of data, we submitted it to a robust variant of the Mahalanobis distance based on the minimum covariance determinant, the Mahalanobis-MCD,^46^ involving use of the MCD75 method, which employs subsamples of size h = n/2 and a breakdown point of 0.001. This method identified 719 multivariate outliers who were excluded from analyses.

The final sample consisted of 1,469 participants with ages varying between 18 and 69 years old (mean [M] = 24.40; standard deviation [SD] = 8.51), the majority being women (89.7%), with high school education (52.6%), white (54.7%), single (71.1%), and from the southeast region (47.5%). The information collected about mental health indicated that 24.2% declared psychiatric treatment, 28.2% psychological treatment, and 25.5% psychiatric diagnoses. An epidemiological study conducted in the megacity of São Paulo (Brazil) found a prevalence in the general population of 2.7% for cluster B, including BPD.^47^ Based on these findings, we can estimate that the sample of this study should have at least 13 people with BPD. More specifically, as previous studies indicate a prevalence of BPD between 1.1 and 3% in community samples,^12,13^ we can assume from 16 to 44 people with BPD in our sample. Table 1 presents details of the sample demographics.

**Table 1:** - Details of the sample demographics

Demographic/category	n (%)
Sex	
Female	1,317 (89.7)
Male	152 (10.3)
	
Psychiatric diagnosis	
No	1,095 (74.5)
Yes	374 (25.5)
	
Suicide attempt	
No	1,072 (73.0)
Yes	397 (27.0)
	
Suicidal ideation	
No	387 (26.3)
Yes	1,087 (73.7)
	
Ethnicity	
White	803 (54.7)
Brown	462 (31.4)
Black	177 (12.0)
Asian	7 (0.5)
Other	9 (0.6)
	
Level of education	
Elementary school	66 (4.5)
High school	773 (52.6)
Undergraduate	280 (19.1)
University education	210 (14.3)
Graduate	140 (9.5)
	
Marital status	
Single	1,044 (71.1)
Married	316 (21.5)
Divorced	33 (2.2)
Widowed	7 (0.5)
Other	69 (4.7)
	
Brazilian region of residence	
Southwest	698 (24.1)
Northeast	354 (47.5)
South	140 (9.5)
North	129 (8.8)
Midwest	148 (10.1)

### Measures

#### Level of Personality Functioning Scale – Brief Form 2.0 (LPFS-BF 2.0)48

The LPFS-BF 2.0 is a self-report scale for assessing impairments in the global personality pattern, as proposed in Criteria A of the AMPD presented in DSM-5. The LPFS-BF 2.0 consists of 12 items answered on a four-point Likert scale and two impairment-related factors: Self and Interpersonal. Evidence supports the psychometric properties of the LPFS-BF 2.0.^49,50^ Alpha and omega values were Self (α = 0.88; Ω = 0.88) and Interpersonal (α = 0.80; Ω = 0.81).

#### Personality Inventory for DSM-5 (PID-5)51

The PID-5 is a self-report scale that measures 25 facets of maladaptive personality traits described in section III of the DSM-5, which can be combined into five domains. The items are answered on a 4-point Likert scale. Studies support the psychometric properties of PID-5.^51^ The following facets were selected based on DSM-5 section III: Hostility (α= 0.89; Ω = 0.91); Impulsivity (α = 0.92; Ω = 0.92), Risk-Taking (α = 0.85; = Ω = 0.86) Anxiety (α = 0.89; Ω = 0.88), Depression (α = 0.93; Ω= 0.93); Emotional Lability (α = 0.84; Ω = 0.82), and Separation Insecurity (α = 0.89; Ω= 0.90).

#### IDCP-2

The IDCP-2 is a self-report scale developed for the evaluation of pathological personality traits based on prominent literature, composed of 206 items on a 4-point Likert scale, grouped in 12 dimensions and 47 factors. Previous studies support the psychometric properties of the IDCP-2.^52,53^ In this study, we administered ten factors reported in the literature as functioning characteristics of BPDs: Vulnerability (α = 0.79; Ω = 0.81), Anxious Worry (α = 0.77; Ω = 0.77), Anxious (α = 0.81; Ω = 0.81), Depressivity (α = 0.89; Ω = 0.89), Impulsiveness (α = 0.82; Ω = 0.83 ), Risk-Taking (α = 0.84; Ω = 0.84), Self-devaluation (α = 0.92; Ω = 0.93), Deceitfulness (α = 0.86; Ω = 0.87 ) Antagonism (α = 0.86; Ω = 0.87), and Abandonment Avoidance (α = 0.85; Ω = 0.85).

#### PQ-SCID-II54

The PQ-SCID-II is a self-report measure developed to evaluate pathological personality based on DSM-IV. The PQ-SCID-II consists of 121 items answered either yes or no, in which each question refers to a diagnostic criterion for personality disorders. Previous studies support the psychometric properties of the SCID.^55^ In this study, we administered 15 items corresponding to the BPD diagnostic criteria. This study’s alpha and omega values were α = 0.83 and Ω = 0.83.

## Data analysis

We first conducted a descriptive analysis. We separated the sample using two different methods, (a) based on the clinical approach described in DSM-5 Session III and (b) an empirical approach using latent profile analysis (LPA). We employed the LPFS to assess impairment in personality based on DSM-5 (criterion A) and the PID-5 to assess BPD traits (criterion B).^8^ We created three groups: people negative for criterion A and negative for criterion B (healthy; n = 884); people positive for criterion A and negative for criterion B (other PD; n = 437); and people positive for criteria A and B (BPD; n = 187). We used the PID-5 facets to determine the groups for the empirical approach (LPA). We created three groups: lower BPD (n = 536), moderate BPD (n = 686), and higher BPD (n = 247).

We compared the scores obtained by each group in the pathological traits using multivariate analysis of covariance (MANCOVA) with post hoc testing (Bonferroni), controlling for the effect of the variable biological sex. We controlled this variable because BPD is more prevalent in women than men.^8^ We employed the Bonferroni correction using the following formula^56^: p-value_corrected_ = p/H, where p is the standard p cutoff (0.05), and H is the number of hypotheses in the study (12). This procedure generated a p < 0.004, employed in our study. We used partial eta squared as the effect size indicator. The partial eta squared was interpreted as 0.01 (small), 0.09 (medium), and 0.25 (large).^57^

We selected the IDCP-2 factors most discriminant of BPD and created a BPD score. We investigated the intercorrelations among IDCP-2 factors to observe the presence of factor independence. We used the receiver operating characteristic (ROC) to explore the best cutoff for the BPD score and calculate the sensitivity, specificity, true predictive value, negative predictive value, positive probability rates, negative probability rate, and the efficiency test of the scales.^57,58^ We compared the values obtained for the IDCP-2 with those obtained by the PQ-SCID-II to verify the capability of the IDCP-2 compared to a similar measure. We used the formula proposed by Streiner^59^ for samples without known prevalence to calculate the positive and negative predictive values. These procedures were conducted with SPSS version 21.

## Results

Table 2 presents the MANCOVA for the healthy, other PD, and BPD groups. These findings indicate significant differences in the IDCP-2 factors, even after controlling for the effect of biological sex.

**Table 2:** MANCOVA results for the DSM-5-based groups

IDCP-2 factor/Group	M	95%CI		F	p-value	Partial eta squared
		Lower bound	Upper bound			
Vulnerability						
Healthy	1.96	1.92	2.00	445.869	< 0.004	0.38
Other PD	2.62	2.56	2.67			
BPD	3.21	3.12	3.29			
						
Anxious worry						
Healthy	2.40	2.36	2.44	249.099	< 0.004	0.25
Other PD	2.95	2.89	3.01			
BPD	3.34	3.25	3.42			
						
Separation insecurity						
Healthy	1.65	1.61	1.70	176.382	< 0.004	0.19
Other PD	2.15	2.08	2.21			
BPD	2.57	2.47	2.66			
						
Anxious						
Healthy	2.30	2.25	2.35	268.658	< 0.004	0.24
Other PD	2.91	2.85	2.98			
BPD	3.41	3.30	3.51			
						
Depressivity						
Healthy	1.93	1.88	1.98	514.733	< 0.004	0.41
Other PD	2.98	2.91	3.06			
BPD	3.55	3.44	3.66			
						
Impulsivity						
Healthy	1.50	1.46	1.54	268.658	< 0.004	0.27
Other PD	1.82	1.76	1.87			
BPD	2.54	2.46	2.62			
Risk-taking						
Healthy	1.34	1.31	1.38	77.495	< 0.004	0.10
Other PD	1.46	1.41	1.51			
BPD	1.85	1.78	1.92			
						
Deceitfulness						
Healthy	1.50	1.46	1.54	68.589	< 0.004	0.09
Other PD	1.71	1.65	1.77			
BPD	2.07	1.98	2.16			
						
Abandonment avoidance						
Healthy	1.84	1.80	1.89	258.237	< 0.004	0.26
Other PD	2.34	2.28	2.40			
BPD	2.95	2.86	3.05			
						
Antagonism						
Healthy	1.42	1.39	1.46	77.495	< 0.004	0.10
Other PD	1.63	1.58	1.68			
BPD	1.97	1.89	2.05			
						
Self-devaluation						
Healthy	1.89	1.84	1.93	531.679	< 0.004	0.42
Other PD	2.84	2.78	2.91			
BPD	3.52	3.42	3.62			
						
Hopelessness						
Healthy	1.54	1.50	1.59	492.721	< 0.004	0.40
Other PD	2.41	2.35	2.48			
BPD	3.11	3.01	3.21			

95%CI = 95% confidence interval; BPD = borderline personality disorder; DSM-5 = Diagnostic and Statistical Manual of Mental Disorders, 5th edition; IDCP-2 = Dimensional Clinical Personality Inventory 2; MANCOVA = multivariate analysis of covariance; PD = personality disorder.

Differences between groups were obtained controlling for the influence of the biological sex variable.

**Figure 1:**
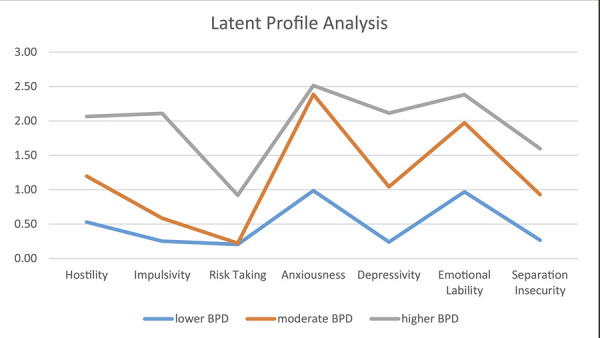
Means of the groups according to latent profile analysis (Personality Inventory for Diagnostic and Statistical Manual of Mental Disorders, 5th edition [DSM-5] [PID-5]). BPD = borderline personality disorder.

The BPD group showed the highest means in all IDCP-2 factors compared to the other groups. The pathological group showed the highest means compared to the healthy group. The effect size ranged between 0.10 and 0.42, mostly interpreted as large.^57^ We conducted an LPA to empirically discriminate groups according to PID-5 facets The correlations between IDCP-2 and PID-5 can be found in Supplementary Table S1. The fit indices for the 3-profile solution were loglikelihood = -11033.775 (4); Akaike information criterion (AIC) = 22127.550; Bayesian information criterion (BIC) = 22286.320; adjusted BIC (aBIC) = 22191.019; Vuong-Lo-Mendell-Rubin likelihood ratio test = p < 0.05; Lo-Mendell-Rubin likelihood ratio test = p < 0.05; bootstrapped likelihood ratio test = p < 0.01; and entropy = 0.82. We chose the solution with three profiles as it presented a better interpretive possibility. Figure 1 shows the groups’ scores for the seven PID-5 facets.

The best interpretive solution was three groups: people showing lower levels of BPD traits (means between 0 and 1 in PID-5 facets) compared to other groups (lower BPD; n = 536); people with moderate BPD trait levels (means between 1 and 2 in most PID-5 facets) (moderate BPD; n = 686); and people with higher levels of BPD traits compared to other groups (means > 2 in most PID-5 facets) (higher BPD; n = 247).

We conducted a second MANCOVA using the LPA groups. The MANCOVA findings indicated that the three LPA groups showed significant differences in IDCP-2 factors even after controlling for the effect of biological sex. Table 3 shows MANCOVA results for the LPA-based groups.

**Table 3:** MANCOVA for the LPA-based groups

IDCP-2 factor/Group	M	95%CI		F	p-value	Partial eta squared
		Lower bound	Upper bound			
Vulnerability						
Lower BPD	1.77	1.72	1.81	537.873	< 0.004	0.42
Moderate BPD	2.45	2.41	2.49			
Higher BPD	3.12	3.05	3.19			
						
Anxious worry						
Lower BPD	2.11	2.06	2.15	518.794	< 0.004	0.41
Moderate BPD	2.95	2.91	2.99			
Higher BPD	3.20	3.13	3.27			
						
Insecurity						
Lower BPD	1.52	1.46	1.58	190.130	< 0.004	0.21
Moderate BPD	2.03	1.98	2.08			
Higher BPD	2.48	2.39	2.56			
						
Anxious						
Lower BPD	1.93	1.88	1.99	529.074	< 0.004	0.42
Moderate BPD	2.95	2.90	3.00			
Higher BPD	3.23	3.15	3.31			
						
Depressivity						
Lower BPD	1.67	1.60	1.73	544.231	< 0.004	0.43
Moderate BPD	2.72	2.66	2.77			
Higher BPD	3.40	3.31	3.50			
						
Impulsivity						
Lower BPD	1.39	1.35	1.43	471.245	< 0.004	0.39
Moderate BPD	1.68	1.64	1.71			
Higher BPD	2.59	2.53	2.66			
						
Risk-taking						
Lower BPD	1.32	1.28	1.36	183.614	< 0.004	0.20
Moderate BPD	1.35	1.31	1.38			
Higher BPD	1.97	1.91	2.03			
						
Deceitfulness						
Lower BPD	1.46	1.41	1.51	110.049	< 0.004	0.13
Moderate BPD	1.59	1.54	1.63			
Higher BPD	2.15	2.07	2.22			
						
Abandonment avoidance						
Lower BPD	1.62	1.57	1.67	391.281	< 0.004	0.35
Moderate BPD	2.27	2.22	2.31			
Higher BPD	2.87	2.79	2.94			
						
Antagonism						
Lower BPD	1.37	1.32	1.41	129.944	< 0.004	0.15
Moderate BPD	1.53	1.49	1.57			
Higher BPD	2.02	1.96	2.09			
						
Self-devaluation						
Lower BPD	1.64	1.58	1.70	573.023	< 0.004	0.44
Moderate BPD	2.60	2.55	2.65			
Higher BPD	3.37	3.28	3.45			
						
Hopelessness						
Lower BPD	1.36	1.30	1.41	519.386	< 0.004	0.41
Moderate BPD	2.14	2.09	2.19			
Higher BPD	3.02	2.94	3.11			

95%CI = 95% confidence interval; BPD = borderline personality disorder; IDCP-2 = Dimensional Clinical Personality Inventory 2; LPA = latent profile analysis; MANCOVA = multivariate analysis of covariance.

Differences between groups were obtained after controlling for the influence of the biological sex variable.

The higher BPD profile showed the highest means in the IDCP-2 factors compared to the other groups. The moderate BPD showed higher means in the IDCP-2 factors compared to the lower BPD. The ηp2 values ranged between 0.13 and 0.44, mostly interpreted as large.^57^ We selected all the IDCP-2 factors to compose the BPD score as they were discriminative in our previous comparisons. We first conducted a Pearson correlation to verify the independence of the IDCP-2 factors. The correlation values ranged between 0.10 and 0.82 (M = 0.43; SD = 0.19), indicating overall independence among factors.

We conducted two ROC curve analyses and generated accuracy indicators to investigate the best cutoff for the BPD score with the DSM-5-based groups and the empirically derived LPA-based groups. We also performed these analyses with the PQ-SCID-II to enable comparison with the results obtained with the BPD score. Table 4 presents the IDCP-2 and PQ-SCID-II results.

**Table 4:** - BPD score and PQ-SCID-II discriminative indicators

BPD score									
Groups	Cutoff	AUC	Ss	Sp	+PV	-PV	+PR	-PR	AC
DSM-5	25	0.96	0.94	0.85	0.66	0.98	6.27	0.07	0.85
LPA profiles	22	0.98	0.96	0.85	0.75	0.98	6.33	0.06	0.89
									
PQ-SCID-II									
DSM-5	9	0.95	0.92	0.81	0.52	0.98	4.80	0.10	0.82
LPA profiles	9	0.97	0.95	0.85	0.80	0.98	6.46	0.03	0.91

+PR = positive probability rates; +PV = positive predictive value; AC = global accuracy.

AUC = area under the curve; BPD = borderline personality disorder; DSM-5 = Diagnostic and Statistical Manual of Mental Disorders, 5th edition; LPA = latent profile analysis; PQ-SCID-II = Diagnostic and Statistical Manual of Mental Disorders, 4th edition – Personality Questionnaire; -PR = negative probability rates; -PV = negative predictive value; Sp = specificity; Ss = sensitivity.

We chose the cutoff with the best relationship between sensitivity and specificity for screening scales,^60^ i.e., prioritizing sensitivity over specificity. We employed Streiner’s formula^59^ for samples without prevalence information to calculate the positive and negative predictive values. The indicators demonstrated the BPD score’s ability to identify the groups, based on the DSM-5 and LPA, mainly to identify the positive cases correctly. The global accuracy indicated that the BPD score correctly identified 85% of participants in the group based on the DSM-5 and 89% based on the LPA. The BPD score showed similar indices to the PQ-SCID-II for discriminating both groups based on DSM-5 and LPA profiles. For instance, the PQ-SCID-II correctly identified 82% (DSM-5-based) and 91% (LPA-based).

## Discussion

Extreme levels of BPD traits significantly impair various aspects of patients’ lives, leading to difficulties in interpersonal relationship,^61^ poor work performance,^1^ suicidal tendencies, and substance abuse.^62^ These impairments directly jeopardize the quality of life of individuals with pronounced BPD traits, emphasizing the necessity of early screening and identification. Our study, grounded in prior IDCP-2 research and BPD literature, examined the potential of IDCP-2 factors to identify individuals with elevated BPD traits in a general population sample. The findings supported our hypothesis that IDCP-2 factors could distinguish individuals with heightened BPD traits. However, the hypothesis regarding the most discriminative factors was partially sustained, with Vulnerability, Depression, Impulsiveness, Self-devaluation, and Hopelessness emerging as the most discriminatory factors in the AMDP-based group (DSM-5-TR), and Vulnerability, Anxious Worry, Depressivity, Self-devaluation, and Hopelessness demonstrating superior discriminatory power within LPA profiles. This research underscores the pivotal role of specific personality traits in understanding BPD, offering insights into potential targeted interventions and support strategies.

The IDCP-2 factors exhibited robust discriminative ability for identifying individuals with elevated BPD traits in both sample division procedures, based on the AMPD^8^ and empirically derived via LPA. Outstandingly, this discriminative capacity remained statistically significant and yielded large effects,^57^ even after controlling for the influence of biological sex and applying the Bonferroni correction.^56^ Consistent with expectations,^8,63^ the biological sex variable emerged as a significant factor in nearly all between-group comparisons, affirming its substantial impact on BPD traits. These findings suggest that the IDCP-2 factors effectively capture the variance in levels of typical BPD traits as reported in the existing literature.^8,26-29^

In the context of the DSM-5-based group, the most discriminating traits included Vulnerability, Depression, Impulsiveness, Self-devaluation, and Hopelessness, while for the LPA-based group, the factors were Vulnerability, Anxious Worry, Depressivity, Self-devaluation, and Hopelessness. These outcomes align with prior research investigating the IDCP-2 factors’ discriminative potential for identifying BPD.^7,38^ Those studies identified Hopelessness, Vulnerability, Anxious Worry, Impulsiveness, and Risk-Taking as the most distinguishing factors, with only Risk-Taking not emerging among the top factors in our study. This factor denotes a more adventurous and risk-prone style,^5^ a characteristic recognized and documented as central in the pathological pattern of BPD.^8,26-29^ As noted earlier, the lower discriminative power of Risk-Taking in our study may be attributed to social undesirability linked to behaviors associated with this trait. This aspect might have led to reduced variability in participants’ responses, consequently impacting its expected discriminative capacity compared to other traits.

We derived a BPD score from the IDCP-2 factors, leveraging our findings. Notably, the BPD score demonstrated excellent performance, as evidenced by the AUC results, aligning with established standards,^64,65^ in effectively distinguishing between the DSM-5 and LPA groups. In the DSM-5-based group, the BPD score exhibited robust performance, accurately identifying 94% of positive and 85% of negative cases. Similarly, within the LPA group, the BPD score excelled, correctly identifying 96% of positive and 85% of negative cases. These results fall within the expected range for PD screening tests, as Merlatin et al.^66^ indicated, where values in the literature typically vary between 92 and 94% for identifying positive cases and between 79 and 85% for identifying negative cases. Moreover, our findings revealed a favorable balance between false negatives and false positives in the BPD score’s performance, aligning with the desired attributes of screening scales.^62^ Specifically, screening scales should be designed to produce more false positives than false negatives, ensuring that individuals with clinically relevant impairments are not erroneously overlooked, thereby ensuring they receive the necessary treatment and support.

We compared the BPD score with the PQ-SCID-II and found that both scales exhibit comparable abilities to distinguish the DSM-5 and LPA groups, with promising indicators.^62^ Despite the BPD score containing more items, it performs on par with the PQ-SCID-II. However, the distinct advantage of the BPD score lies in its capacity not only to screen for BPD but also to pinpoint the specific traits in which the patient exhibits significant changes, providing valuable insights into the clinical profile.

Our study has methodological limitations that warrant consideration when interpreting and extending its findings. Our sample was drawn exclusively from the general population, which may limit the generalizability of the results to clinical populations. Although we used two distinct procedures to identify individuals with BPD traits, one of which employed the PID-5 as an external criterion, inclusion of clinically diagnosed BPD patients using diagnostic interviews would enhance the robustness of group composition. We recommend that future investigations include individuals with confirmed clinical BPD diagnoses to bolster the clinical relevance of findings. Additionally, examining the discriminative capacity of IDCP-2 factors to differentiate BPD from other personality disorders represents an important avenue for future research.

Our results support the clinical utility of the BPD score for identifying individuals with high levels of BPD traits. This score serves as a valuable tool for clinical screening and offers a comprehensive profile of a patient’s presentation across 12 typical BPD traits. Notably, our findings indicate the presence of two distinct cutoffs for identifying BPD, contingent on group categorization. To adopt a more conservative approach, we recommend employing a cutoff score of 22 on the BPD scale. This threshold can effectively highlight patients warranting clinical attention and further assessment for potential BPD-related concerns.

## Supplementary Material

Supplementary Material
