# Supplementary material for: Identifying borderline traits in a Brazilian community sample using the Dimensional Clinical Personality Inventory 2 factors
Source: Trends Psychiatry Psychother. 2025 Sep 18;47:e20240871. doi: 10.47626/2237-6089-2024-0871 (PMC12962370; doi:10.47626/2237-6089-2024-0871)
Supplement: Supplementary Material [file 2238-0019-trends-47-e20240871-suppl01.pdf]

http://doi.org/10.47626/2237-6089-2024-0871

Supplementary Table S1 - Pearson correlations between the IDCP-2 factors and the PID-5 facets

	Vulnerability IDCP-2	Anxious worry IDCP-2	Anxious IDCP-2	Depressivity IDCP-2	Impulsiveness IDCP-2	Risk-taking IDCP-2	Deceitfulness IDCP-2	Dep2	Abandonment avoidance IDCP-2	Hostility PID-5	Impulsivity PID-5	Risk-taking PID-5	Anxiousness	Depressivity	Emotional lability	Separation insecurity	SCID_border
Vulnerability	1																
Anxious worry	0.560*	1															
Anxious	0.553*	0.717*	1														
Depressivity	0.579*	0.542*	0.521*	1													
Impulsiveness	0.562*	0.339*	0.290*	0.397*	1												
Risk-taking	0.349*	0.105*	0.125*	0.209*	0.565*	1											
Deceitfulness	0.355*	0.141*	0.130*	0.216*	0.471*	0.492*	1										
Abandonment avoidance	0.487*	0.669*	0.478*	0.484*	0.390*	0.160*	0.194*	1									
Antagonism	0.415*	0.155*	0.182*	0.265*	0.507*	0.538*	0.705*	0.189*	1								
Hostility	0.649*	0.337*	0.418*	0.425*	0.499*	0.322*	0.385*	0.296*	0.497*	1							
Impulsivity	0.565*	0.342*	0.326*	0.406*	0.763*	0.505*	0.403*	0.402*	0.445*	0.539*	1						
Risk-taking	0.326*	0.085*	0.107*	0.199*	0.524*	0.835*	0.460*	0.140*	0.535*	0.360*	0.544*	1					
Anxiousness	0.493*	0.708*	0.776*	0.496*	0.258*	0.073*	0.095*	0.469*	0.137*	0.393*	0.333*	0.090*	1				
Depressivity	0.540*	0.436*	0.424*	0.766*	0.383*	0.248*	0.213*	0.429*	0.268*	0.415*	0.467*	0.267*	0.437*	1			
Emotional lability	0.520*	0.442*	0.457*	0.413*	0.361*	0.166*	0.124*	0.412*	0.133*	0.459*	0.419*	0.187*	0.542*	0.377*	1		
Separation insecurity	0.371*	0.590*	0.383*	0.358*	0.313*	0.101*	0.144*	0.755*	0.117*	0.245*	0.387*	0.124*	0.455*	0.377*	0.379*	1	
SCID_border	0.734*	0.473*	0.470*	0.592*	0.578*	0.374*	0.395*	0.437*	0.442*	0.656*	0.621*	0.385*	0.472*	0.581*	0.525*	0.385*	1

IDCP-2 = Dimensional Clinical Personality Inventory 2; PID-5 = Personality Inventory for Diagnostic and Statistical Manual of Mental Disorders, 5th edition (DSM-5).
* Correlation is significant at the 0.01 level (2-tailed).
